# Effects of Nondipping Blood Pressure Changes: A Nephrologist Prospect

**DOI:** 10.7759/cureus.42681

**Published:** 2023-07-30

**Authors:** Elmukhtar Habas, Raza A Akbar, Gamal Alfitori, Khalifa L Farfar, Eshrak Habas, Nada Errayes, Aml Habas, Aisha Al Adab, Amnna Rayani, Nagat Geryo, Abdel-Naser Y Elzouki

**Affiliations:** 1 Internal Medicine, Hamad General Hospital, Doha, QAT; 2 Internal Medicine, Alwakra General Hospital, Doha, QAT; 3 Internal Medicine, Tripoli University, Tripoli, LBY; 4 Medical Education, University of Lincoln, Lincoln, GBR; 5 Renal and Dialysis, Tripoli Pediatric Hospital, Tripoli, LBY; 6 Pulmonary Medicine, Hamad General Hospital, Doha, QAT; 7 Hemato-Oncology, Tripoli Pediatric Hospital, Tripoli University, Tripoli, LBY; 8 Medicine, Hamad General Hospital, Doha, QAT; 9 Internal Medicine, Hamad Medical Corporation, Doha, QAT

**Keywords:** abnormal bp pattern-related mortality, kidney and nondipping bp, target organ damage, htn, altered circadian bp rhythm, nocturnal bp reverse dipping, ambulatory blood pressure monitoring

## Abstract

Blood pressure (BP) variations depend on various internal, environmental, and behavioral factors. BP fluctuations occur both in normotensive and hypertensive people. Although it fluctuates over the 24-hr day and night, the morning BP increases after waking up and declines throughout sleep. It is typical for BP to decrease by 10% to 20%, while sleeping, known as dipping BP. However, if there is no decrease in nighttime mean systolic BP or a drop of less than 10 mmHg, it is called nondipping BP. Conversely, reverse dipping BP means an increase in mean systolic BP instead of a drop during the night. Reverse dipping is observed in hypertension (HTN), diabetes mellitus (DM), chronic kidney disease (CKD), and obstructive sleep apnea (OSA) syndrome. The introduction of ambulatory BP monitoring (ABPM) led to the emergence of identifying normal and elevated BP patterns. Non-dipping BP increases the risk of cardiovascular system (CVS) complications such as left ventricular hypertrophy, proteinuria, glomerular filtration rate (GFR) reduction, and CKD progression. A loss or blunting of the normal BP profile is recognized as a deleterious variant, and restoring abnormal BP patterns has been reported to significantly impact end-organ damage, morbidity, and mortality.

In this non-systematic clinically-oriented, comprehensive review, we aim to update the BP variables and the pathophysiology of nondipping BP and point out the areas which need more investigation from a nephrology perspective because the nondipping BP increases the risk of proteinuria, GFR reduction, and CKD progression. A literature search of PubMed, Google, EMBASE, and Google Scholar was conducted. Checks of selected papers and relevant reviews complemented the electronic search.

With improved BP measurement methods, the physiology of BP profile variations is readily detectable during the day and night. A nondipping BP profile is a distinct BP pattern that may have significant end-organ damage effects and therapeutic importance for nephrologists.

The pathophysiology of the nondipping BP variant must be clarified to prevent complications, and further investigations are required. Furthermore, there is debate about the best BP index to utilize: systolic BP, diastolic BP, mean arterial pressure, or a mixture of all. All these areas are important and need new research projects.

## Introduction and background

Chronic kidney disease (CKD) is a widespread and significant illness with a global public health impact. Diabetes mellitus (DM) and hypertension (HTN) significantly affect CKD prevalence [1]. The increasing incidence of HTN and DM and the aging society significantly contribute to the rise in CKD patients [2]. Previous epidemiological studies showed that the risks of cardiovascular diseases (CVDs) and end-stage renal disease (ESRD) increase in CKD [3]. Several risk factors affect renal function in CKD patients [4], and high blood pressure (BP) is often considered a significant cause and a predictor [5]. BP control in CKD patients is crucial to prevent CVD, the progression of CKD, and ESRD development [6,7]. However, determining BP is still up for question; notably, the accuracy and reproducibility of traditional clinical BP measurement methods are used [8].

The previously reported studies showed that BP records taken outside of clinic settings through ambulatory BP monitoring (ABPM) or at home had a more significant correlation with the frequency of CV events and the consequent deterioration in kidney function [9]. Not only does ABPM enable the identification of white-coat and masked HTN, but it also permits the categorization of daily BP profiles or patterns [10,11]. Furthermore, abnormal nocturnal BP measurements and patterns assessed by ABPM had more harmful effects than high office BP readings [10,11].

Normally, there is a 10%-20% reduction in BP at night, which is a physiological variation [12]. Individuals who have a decline in the nighttime BP are called “dippers”, while those without such a drop in BP during the night are recognized as “non-dippers” [13]. The nondipper BP profile is frequently witnessed in CKD patients [11,14]. Clinical observational reports demonstrated that the nondipper BP profile is frequently complicated by end-organ, such as kidney damage. Renal damage manifests with increased urinary albumin excretion [15], which is a strong predictor of the progression of renal insufficiency [16]. Normotensive non-dippers may develop CKD because the nondipping BP profile precipitates atherosclerosis in the renal arteries in IgA nephropathy [17]. The target BP of ABPM has not yet been determined because of the paucity of appropriate clinical data, especially from interventional clinical studies using ABPM-based controls. Furthermore, it is not yet clearly proven that the normotensive nondipper pattern is associated with CKD progression.

This is a comprehensive non-systematic review article. The institute’s ethical committee has deemed obtaining ethical approval for this type of work unnecessary. A literature search was performed in PubMed, Google, EMBASE, and Google Scholar. Checks of selected papers and relevant reviews complemented the electronic search. Studies were recognized by crossing the following keywords and terms: ambulatory BP, nocturnal BP, reverse dipping, inverted dipping, altered circadian BP rhythm, HTN, target organ damage, kidney and HTN, kidney, and dipping BP, abnormal BP pattern-related mortality, and other phrases.

## Review

Blood pressure physiology

The autonomic nervous system (ANS), particularly sympathetic nervous system (SNS) activity, is the primary determinant of circadian changes in BP. However, other neurohormonal systems that control BP also affect circadian rhythm, suggesting their involvement in diurnal fluctuations in BP [18]. According to numerous research, increased sodium consumption and salt sensitivity are linked with the decrease or even inversion of some participants' typical nocturnal dipping [19,20]. Fukuda et al. contend that an impairment in the daytime renal sodium excretion is the underlying reason for the nondipping pattern of the night BP [21]. Therefore, the BP rises during nighttime to encourage sodium excretion to maintain a 24-hour sodium balance. The diminished ability to excrete sodium may be brought on by either a decrease in renal function, as seen in those with low an estimated glomerular filtration rate (eGFR), or a rise in sodium reabsorption in the tubules, for instance, in primary aldosteronism [22]. Several modest investigations of selected salt-sensitive and salt-resistant individuals provide the main evidence for the theory connecting the dipping pattern to the ability to excrete sodium at night [20]. A 24-hour BP reading, renal function, and day with overnight urine samples revealed elevated urinary sodium in normal and hypertensive African-American people [23].

Urine sodium concentration decreases due to V2-antidiuretic hormone (ADH) receptor increased activity in the collecting duct of the neuron by ADH. Inhibition of this action leads to increased sodium excretion. During the day, the plasma ADH level is higher than at nighttime, leading to more sodium reabsorption by nephron tubules and collecting ducts, leading to a higher body content of sodium during the daytime. ADH and other vasopressor hormone plasma levels decrease during nighttime, leading to more loss of sodium and the consequent dipping of BP. This theory was supported by a clinical study by Bankir et al. [24]. Another study evaluated the effect of tolvaptan (a selective vasopressin receptor antagonist) on the parts of nephron which handle water and sodium equilibrium. Systemic hemodynamics revealed that the reduction of water excretion by the nephrons is dose-dependent. In contrast, the reduction in sodium excretion was dose-independent, partly through an AVP-dependent mechanism [25]. Thus, tolvaptan counteracted the kidneys' ability to diminish sodium and water loss caused by nitric oxide inhibition. Tolvaptan's failure to reduce the excretion of urine by the aquaporin-2 channels may be due to the high plasma ADH concentration [25]. Therefore, activation of V2 receptors enhances salt and water reabsorption while not dependent on vasopressin concentration alone. There still seems to be a lack of clear understanding of these mechanisms, which necessitates further research in this area.

Another suggested theory for the dipping of BP at sleeping is position. While sleeping, humans lie flat, which logically causes BP to drop. Furthermore, during sleeping, autonomic nerve activity alters by decreasing sympathetic and increasing parasympathetic, affecting BP. Moreover, blood flow redistribution and decreased blood pooling in lower limbs compared with an upright position increase kidney blood flow, eGFR, sodium filtration, and excretion in lying down. Although these are suggestive BP dipping mechanisms at night, further investigatory studies are required to prove or disprove them.

Blood pressure measurement and blood pressure types

Historically, BP measurement was usually done by physicians. As time evolved, the nurses and even the patients started to measure it, using oscillometric automatic BP machines. The ABPM allows BP measurement while the subject is awake or asleep. ABPM is usually taken every 20-30 minutes throughout the daytime and every hour during sleeping [26]. The means of the BP readings at night and day are calculated. The mean of the daily readings is known as “day BP” and at night while sleeping, called “night, nocturnal, or BP during sleep” readings. The mean percentage of systolic BP reduction by about 10-20% during sleep is called dipping BP. However, people who have < 10% drop of their BP are labeled as nondippers. In some subjects, measuring the BP at the doctor’s office can be less informative and nonconfirmatory without ambulatory monitoring. ABPM might be a superior BP recording method than office readings for certain BP patterns including masked and white-coat HTN.

ABPM seems to be a more reliable predictive method and the best for the prediction of death as compared to the clinic BP measurement [27]. The ABPM may define the physiological status, assess the efficacy of the antihypertensive medications, and detect white-coat, masked, and uncontrolled masked HTN [8]. The 2018 Guidelines for the Management of Arterial HTN by the European Society of Cardiology (ESC) and the European Society of Hypertension (ESH) [28] recommended the adoption of ABPM at least once for better risk stratification of HTN. In contrast to regular dippers, non-dippers and reverse dippers (higher nocturnal BP than daytime) have a higher risk of CVD and a worse prognosis. In diabetic individuals, non-dipping and morning blood surges occur around 40-50% of the time [29].

BP phenotypes are detected from the discrepancy between ABPM and office BP readings. ABPM is acknowledged as a gold standard for assessing a "real" hypertensive state. Additionally, ABPM is the sole method for evaluating aberrant circadian BP patterns, such as the nonexistence of a physiological nocturnal BP decline [7,28]. It is well documented that the sturdier predictor of CV and kidney disease outcomes is ABPM than office BP in individuals with CKD [30-32]. In CKD patients, nondipping status is linked to a bad prognosis for their renal and CV systems [30]. Regardless of the patient's nocturnal dipping status, current recommendations support strengthening BP therapy in above-goal ambulatory BP patients. However, the targeted ABPM reading in the nondipping BP profile is poorly documented. Understanding and identifying the risk of this phenotype is necessary before treating them [33].

Combining office and out-of-office BP values, such as self-measured home BP or 24-hour ABPM, provided an accurate assessment of the BP status over the past four decades. Its use has become more frequent in clinical and research settings [34]. The combined office and home or 24-hour ABPM have indicated four types of HTN. Sustained normotension (i.e., normal BP inside and outside the physician or nurse’s office), sustained HTN (i.e., elevated in-office and out-of-office BP), white-coat HTN, also known as isolated clinic HTN (i.e., elevated BP in the office and normal outside the office), and masked HTN (normal BP inside but elevated outside the clinic office). Significant differences exist between these BP patterns in frequency, demographic and clinical traits, and also in the burden of subclinical organ damage, and the risk of CV complications and death [35,36].

ABPM also allows one to assess day-night BP fluctuation since it provides a considerably greater number of BP readings over 24 hours than office and home BP readings. Most people with normal and high BP readings have a BP circadian rhythm. Nocturnal BP levels are 10% to 20% lower than daytime due to the drop in sympathetic tone and the concurrent rise in vagal activity during sleep. In the past, people with aberrant circadian rhythms (nondippers) have often been defined as having nocturnal BP drops lower than 10% [37,38]. Physical exercise has different effects on both systolic and diastolic BP. The systolic BP increases linearly with higher activity levels, but diastolic BP tends to drop. Therefore, dipping categorization might change according to the BP index [39]. The majority of ABPM monitors measure BP oscillometrically. The most accurate measurement is made with this method instead of relying on measuring systolic or diastolic BP. Therefore, employing mean arterial pressure as the BP indicator for dipping status categorization can be suggested. Secondly, it might be argued that "nocturnal" should be changed to "sleeping" in the definition. If a person does not sleep at night, it is unlikely that their BP will decrease. Night shift workers are a good example, in whom the dipping pattern transforms into a nondipping pattern over the first 24 hours of the night shift. Over the following days, the nondipping pattern gradually returns to a dipping pattern. These participants show a drop in their BP readings during daytime sleep [40].

Several combinations of approaches are available to define the ‘sleeping time’. Using diary card entries is an uncomplicated strategy. Some people prefer a limited timeframe to describe the evening, such as midnight to 6 AM, eliminating overlapping times when patients may be awake or asleep [41]. This maneuver has a negligible impact on non-dippers' nighttime dipping proportion as they have a far lower morning BP spike than dippers [42]. However, people identified as borderline dippers via other techniques could be identified as nondippers using other techniques such as ABPM. Utilizing activity and posture monitoring, a more contemporary technique, is entirely accurate, particularly when paired with the diary card input technique [39]. The binary distribution is a preferred term over a continuous term. We could not identify literature evidence to prove that the 10% cutoff point discriminates more than the other nearby cut-off BP readings. However, the 10% cutoff is arbitrary, but it is simple to apply and seems helpful so far, differentiating dippers from nondipper individuals [43].

A nondipping BP pattern's clinical use hinges on consistency from one instance to the next. Manning et al. revealed that only 54% of 79 untreated HTN and normotensive subjects could be reliably categorized as dippers [44]. In other research, 170 hypertensives had two ABPMs that were spaced by more than one year, and it was found that at least 40% had altered their dipping status. It should be stated that while the recordings were made when patients were not receiving antihypertensive medication, participants received BP-lowering medicines between the two measures. Reported findings were more encouraging [45]. Repeated ABPM was conducted on 65 newly diagnosed untreated HTN. After the repeated measurements, 12% of the patients' nocturnal dipping patterns changed from nondipping to dipping, remaining unaltered in 82% of the patients. Additionally, dipping status seemed more repeatable in trials when daily physical activity was assessed objectively [46]. One rationale is that individuals are less inclined to adhere to research procedures when a diary monitors their behavior. Additionally, they probably act similarly in consecutive ABP measures. Alterations in body posture from one night to the next may impact repeatability [47]. If the cuff is fastened to the upper arm or the forearm, it may produce a difference of roughly 12 to 14 mmHg while participants sleep on their sides or backs [48]. Activity and posture affect the ABPM. Rectifying the arm position effect on BP variations during repeated recordings using these devices is feasible. However, single research reported that correcting variations in body posture overnight did not enhance the repeatability of dipping status [49].

Physiology of dipping blood pressure

At the start of sleep, recognizable changes occur in autonomic and endocrine activities, including a decrease in sympathetic outflow to the heart and muscle, vascular bed, and a decrease in renin-angiotensin system activity [50,51]. Parasympathetic activity toward the heart also rises while sleeping [52]. According to previous studies, altering the baroreflex set points to low BP values causes nocturnal BP drops [51,53]. It is still unclear whether it is an active process, just a sequence of physical inactivity, or sleep-related exposure to outside stimuli. This area needs further studies to assess these effects.

Phenylephrine is an α-adrenoceptor agonist which does not cross the blood-brain barrier to counteract the natural decline in sympathetic activity due to external stimuli [54]. The BP drop that continues for three hours after physical activity refutes the notion that baroreflex mechanisms serve to slow down sudden fluctuations in BP. Resetting was described as an adaptive modification of the baroreceptors' function. Due in part to its capacity to reset, early research suggested that the baroreflex arch did not significantly affect long-term control [55].

The peripheral baroreflex arch is controlled by a central nervous system-determined set point [56]. Because alertness always obscures or continually alters the arterial baroreflex activity, the nocturnal sleep phase is the only possible time that permits the measurement of this centrally directed set point. This concept is consistent with recent animal studies that have brought attention to the notion of a central nerve set point [56]. It is interesting to note that Pattoneri et al. observed that patients with prolonged vegetative traumatic brain injury had sympathetic and vagal abnormalities in the modulation of vasomotor tone, resulting in a nondipping pattern [57]. These results prove the central neuronal hierarchy that controls BP in humans. Integrating several inputs into this system includes endocrine impacts through body fluid-electrolyte concentration, osmolality effects, and CV pressure responses [58,59]. These findings might indicate that a super-ordinated central neural regulation of baroreflex function also occurs in humans.

An experiment focused on how sleep affects the efferent part of the sympathetic system activity in the vascular muscle bed, causing vasoconstriction. One potentially implicated route in the circadian BP profile is primarily addressed by maintaining nocturnal BP increased to non-physiological ranges using intravenous vasoconstrictors [58]. However, it was impossible to provide comparable precision, especially cardiac baroreflex function [59]. Microneurographic techniques used to measure baroreflex gain depict a skewed input-output connection between blood vessel smooth muscle sympathetic nerve activity. The pharmacological effects of this connection could influence the BP pattern [60]. However, this simplification was purposefully made as a scientific pattern delving into significant factors regulating BP while sleeping. Whether the results are general or related to the substances used is unclear. Changing baroreflex set-point of the vascular beds was seen at rest and confirmed across a wide range of BP readings during the infusion of differently-acting drugs (nitroprusside and phenylephrine). It is considerably more plausible that a novel element of nighttime BP physiology that suggests long-lasting consequences throughout the daytime was revealed. However, this precept must be verified in other research within larger populations.

The relevance of the rennin-angiotensin system in BP control and permanently resetting the baroreflex has previously been acknowledged [61,62]. Heart rate (HR) variability, serum electrolyte concentration, and osmolality did not alter morning BP. However, plasma angiotensin II and renin levels were much lower than the night prior to the phenylephrine infusion. The baroreceptor set point deviated southward due to selective angiotensin-receptor inhibition [61]. As a result, one reason for the baroreflex curve change might be the low angiotensin II levels in the morning [63]. It is conceivable that the drop in renin and angiotensin levels reflects the passive effects of nondipping, and it has a non-significant regulatory impact on baroreflex function; however, this hypothesis cannot be proven with the currently available data, hence the need for new research in this domain.

Perspectives of blood pressure physiology

Nocturnal non-dipping control significance indicates that BP dipping during overnight sleep is a process of active downregulation. Any violation of this process, such as extrinsic nondipping, is vigorously retaliated against by the body, even causing BP levels to drop the next day without a significant autonomic and sympathetic response imbalance. The research emphasizes the significance of coordinated central neurological systems, which, via baroreflex mechanisms, actively reduce BP during sleep. This presents a fresh viewpoint on the function of sleep-in controlling BP under physiological and pathological circumstances. To clarify the mechanism(s), new studies are required on the mechanism(s) of the physiology of BP changes during daytime and sleeping time.

Blood pressure variances

Disruptions in the BP rhythms increase the risk of diseases associated with hypertension independently of the actual BP readings. However, this technique needs to consider the daily regularity of BP change. Clinical guidelines are being improved to include using ABPM to acquire data over extended periods of activity and rest while doing daily activities [28,64]. This is done in recognition of the fact that the circadian pattern of daily BP is essential information for diagnosing and managing HTN. BP rhythm is endogenously created without external timing signals and cycles for a full day. It continues to exist even during studies that impose 20- or 28-hour cycles of activity, sleep, and eating cycles [65].

In healthy people, the daily rhythm of the BP is defined by a morning rise after waking up, which peaks throughout the daytime, followed by a nighttime dip. In 1988, the term "nondipper" was developed to refer to HTN patients with a diminished nocturnal decline who also had an elevated risk of stroke [66]. Nondipping, now more precisely defined as a nocturnal BP decrease of < 10% compared with daily readings, is frequently used to refer to unusually rising BP at rest. Nocturnal HTN diagnosis is based on absolute BP levels rather than changes from the daytime reading. Referring to the American Hypertension Association (AHA), 2017, BP > 110/65 mmHg [6] may be quantitatively reliable [67] but may not always indicate an abnormal endogenous BP pattern. Notably, nighttime HTN and nondipping BP enhance risk, with individuals with both characteristics showing the poorest profile [68]. Various CVDs, such as left ventricular (LV) hypertrophy [69], atherosclerotic plaques [70], congestive heart failure [71], vascular dementia [64], and myocardial infarction, are all linked to nondipping [72]. Furthermore, microalbuminuria indicates an early kidney involvement in nondipping HTN [73]. Nocturnal BP or nondipping was linked to all causes of mortality and aggregate CVD outcomes [72]. The most extensive meta-analysis of hypertensive patients’ research used the ABPM BP measurement method, revealing that nondipping independently predicts CV events [74]. The risk of CVD is doubled by masked nocturnal HTN [75].

**Figure 1:**
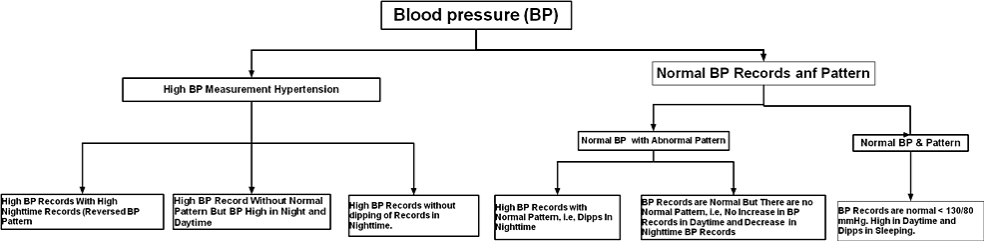
Summary of Blood Pressure Variants.

ABPM usage in a primary care setting revealed nocturnal HTN in 30% of individuals with normal office BP readings [76], suggesting that this kind of masked high BP may be common. Its appearance early in the diabetes progressive course anticipates the onset of nephropathy and appears in most individuals with CKD [14]. Significantly, nondipping precedes and forecasts renal disease development [77]. A crucial information gap about the cycles of normal BP physiology hampers the therapy for HTN to improve the circadian BP pattern; however, it is easier said than done. Day-night fluctuations in activity [78] and HR [79] are significant contributors, among other variables, to BP dipping-non-dipping patterns. The BP variance types are illustrated in Figure 1.

Dipping blood pressure

Most normal individuals possess a dipping BP pattern in which their nocturnal BP is 10-20% less than their daytime BP. Over the last three decades, dipping patterns in hypertensive patients have been intensively examined. Comparing the overall mortality in hypertensive dippers and nondippers verified that the dippers had a decreased risk of overall mortality, which was reinforced by the lack of heterogeneity across trials. Moreover, comparing the risk of stroke among the two groups demonstrated no significant differences; the same was true for subgroup analysis of ischemic strokes [80]. Additionally, the risk of overall mortality and stroke was not substantially different between dippers and nondippers; however, reverse dippers had an increased stroke risk [80].

Decreased dipping and nondipping patterns indicate increased CV risk and HTN-associated organ damage [23,81,82]. The predictive significance of nondipping, reverse dipping and excessive dipping in CV morbidity and death was verified by a comprehensive meta-analysis of 17,312 treated and untreated hypertensive people. Results supported that frequent treatment reduces cardiovascular morbidity and death by restoring the natural dipping pattern [74]. However, recent studies in untreated hypertensive individuals indicate that most surrogate markers of CVD, such as left ventricular hypertrophy, carotid media layer thickness, arterial stiffness, and endothelial dysfunction, are comparable between dippers and non-dippers [83-86]. Several clinical and methodological factors may nullify the predictive usefulness of nondipping in untreated HTN. Such considerations include poor repeatability of the dipping status, the likelihood of very low daytime BP producing a nondipping phenotype without high values of overnight or 24-hour BP, and the confounding influence of associated morbidities [87].

Mechanism of blood pressure dipping during sleep

The natural drop in BP during sleep is due to inactivity and sleep. This reduction may be countered by arguing that sedentary lifestyles and low sleep quality are to blame. It has been hypothesized, for instance, that those with a higher CV event risk are also more likely to be identified as non-dippers because they are less active throughout the day [88]. It was suggested that insufficient daily exercise and deprived sleep are to blame for the non-dipping phenomena, although this theory is contested. First, a study comparing dippers and non-dippers finds that their daytime BP is comparable [89]. Second, patients who report having high sleep quality in their diaries may also have non-dipping BP [89]. Third, non-dipping is associated with various clinical problems that have a nonsignificant effect on daytime activity and sleep quality. Regarding the hemodynamics at play here, overnight systemic vascular resistance is either the same as daytime systemic vascular resistance or is significantly enhanced [49]. Decreased HR contributes to lower cardiac output at night, whereas stroke volume is unaffected [49]. Differences in cardiac output and systemic vascular resistance between day and night have been examined in several studies [49,76,90]. There seems to be a lack of consistency in research results. Thus, a lack of a nighttime decline in BP might be due to a reduced nocturnal reduction in cardiac output, an exaggerated change in systemic peripheral resistance, or both. The difference between night and day readings of BP is primarily because changes in posture and daily activities highly impact the diurnal variations in cardiac output and peripheral resistance [39].

The alteration in systemic activity of the autonomic nervous system during sleep is primarily due to a decrease in parameters such as sympathetic and increased parasympathetic activities, HR and cardiac output. When autonomic dysfunction occurs, BP does not drop as it should during sleep and may even rise throughout the night (nocturnal HTN) [91]: concomitantly, leading to conceding of the SNS, and much blood pools in the legs and feet when the body is upright. Due to diminished renal perfusion caused by the upright position during the day, the kidneys retain fluid throughout the day. Due to decreased autonomic function, the baroreflex cannot prevent an increased stroke volume, cardiac output, and BP caused by lying flat. The pathophysiology of non-dipping may include excessive extracellular fluid due to the associated renal dysfunction, and increased aldosterone and cortisol hormones are commonly seen in patients with this condition. Studies showed that the nondipping BP might be transformed into a dipping BP profile in sodium-sensitive hypertensives by following a sodium-restricted diet or diuretics and in diabetics using Thiazolidinediones [92]. Hence, the underlying mechanisms of the BP dipping phenomenon are likely due to the decreased physiological, physical, and mental activities.

Nondipping blood pressure

Although it is debatable, there is mounting evidence that individuals with essential HTN and nondipping BP probably have more risk of target organ damage [93]. A nondipping BP profile is distinguished by a BP decrease of < 10% at night, associated with the rapid deterioration in renal function in adulthood. The nondipping BP pattern is common in children. However, in a cohort study of children with CKD, Bakhoum et al. observed that nondipping is not linked to ESRD, a drop in eGFR, or a change in proteinuria level [90].

The nondipping BP profile links with a higher rate of CV events and target organ damage. However, there are also concerns about how well ABPM measures the dipping status. Furthermore, nondipping has yet to be given a precise definition. A nondipping profile may have anomalies in extracellular volume and vascular resistance modulation as its pathophysiological mechanism(s). Additionally, variations in day and nighttime activities, sleep quality, and body positioning when sleeping are also involved. The ABPM may define the physiological status, assess the efficacy of the antihypertensive medication, and detect white-coat HTN, masked HTN, and uncontrolled masked HTN. ABPM seems more reliable in predicting prognosis and death than clinical measures [27].

Most people have significantly lower BP at night. A decrease in the typical nocturnal drop should be considered abnormal [94]. Of this abnormality, clinical importance is related to its close association with hypertensive organ damage, its elevated risk for subsequent CV events, and its correlation with clinical conditions like some secondary forms of HTN, renal impairment, and disturbances of the autonomic nervous system [95]. Notably, a nondipping profile could be advantageous in certain situations. For instance, a medically induced nocturnal BP reduction may cause cerebral hypoperfusion in nondipping individuals who already have a compromised cerebral perfusion [96]. A pharmacologically induced reversible non-dipper to a dipper profile may be predicted to reduce CV risk; however, to prove this, further research is required.

Mechanism of nondipping blood pressure

The loss of the kidney’s ability to eliminate sodium during the daytime contributes to a diminished nocturnal BP reduction [97]. Nocturnal nondipping BP profiles link strongly to decreased sodium loss during daytime and the rise in nocturnal BP via the pressure-natriuresis pathway, most likely a compensatory mechanism to enhance salt excretion and preserve sodium balance [98]. Intervention studies indicated that a dipping pattern is restored following salt restriction or diuretic administration, supporting the sodium function in controlling circadian BP rhythm [99]. Normal sustained eGFR is a significant regulator of salt balance; a decrease in the normal overnight BP fall often accompanies a decline in eGFR. The circulating serum levels of cystatin C are less influenced by age, sex, and muscle mass; therefore, cystatin C is more sensitive than serum creatinine to assess the eGFR. Serum cystatin C concentration is substantially higher in essential HTN patients with reversed dipping BP profiles than those with dipping patterns [100].

Relevance of nondipping blood pressure to end-organ damage

Research shows that non-dippers have more pronounced cardiac and extracardiac organ damage than individuals who maintain their nightly BP [101].

As compared to the dippers, the non-dippers have significantly higher rates of left ventricular hypertrophy, increased thickness of carotid intima and media, microalbuminuria, and cerebrovascular disorders [102]. Significantly, a nondipping BP profile links with more severe target organ damage and is an early predictor of CV events in hypertensive and normotensive people [103]. Also, it is well known that a BP pattern in which the BP never drops is linked to diminished renal function [95,104]. In contrast, little data suggests that this pattern also increases the development of renal impairment [105]. Therefore, this area needs further research to assess the pathophysiology of the dipping-non-dipping BP pattern, the pathogenesis of the damage caused and its consequent long-term effects on the end-organs.

Reverse dipping blood pressure

Compared with the daytime readings, reverse or inverted dipping is defined by unaltered or even higher nocturnal BP. Reverse or inverted dipping is a severe, not uncommon, disruption in the circadian BP rhythm. It is often seen as a particularly dangerous BP phenotype [101]. It is frequently recognized in HTN, type 2 DM, CKD, and sleep apnea syndrome. The reversed dipping BP profile is common in sustained HTN and is thought to be mainly due to increased sympathetic tone during sleeping. Augmented sympathetic tone increases total vascular resistance, HR, stroke volume, and cardiac output, increasing BP and reversing the dipping BP profile [106].

The left ventricular mass index in CKD was considerably higher in reverse dippers than in non-dippers and dippers [107]. In CKD, the left ventricular mass index was not substantially greater among reverse dippers than dippers, nondippers, or severe dippers [108]. Among several ambulatory day/night BP profiles, the African American Study of Kidney Disease and HTN Study (Clinical Trial) (AASK Trial) reported slight variations in left ventricular hypertrophy prevalence [109]. Young individuals with a less marked or nonexistent nocturnal BP drop at the first screening had the highest probability of developing coronary calcification 10 to 15 years later [110].

A study has established a substantial link between reversed BP dipping profile and increased urine protein excretion [111]. Proteinuria was 66% higher in reverse dippers than in dippers and nearly four times higher than in severe dippers. Other investigations failed to identify statistically significant differences in proteinuria, defined as continuous or binary variables, between reverse dipper CKD patients and nondipper and dipper patients [112].

The risk of ESRD and CV events related to reverse dipping BP was assessed in 1024 patients. Patients with reversed BP exhibited higher renal and CV events rates than patients with maintained circadian BP rhythm, regardless of 24-hour systolic BP values [111,113]. The increased mortality risk identified among patients with reversible BP was probably due to CV events [101]. In a survey of elderly patients without major CV diseases or other existing morbidities at baseline conducted in a primary care practice in Belgium, reverse BP had prognostic significance in univariable analyses; however, it had lost its predictive value after tuning for confounders, including 24-hour BP [114]. Another study of hypertensive diabetic patients at high risk for CVDs revealed that reverse BP status was independently correlated with higher rates of the composite outcome, including stroke, myocardial infarction, and CV events, during a follow-up period of 2.6 years [115].

The research evaluated the advantages of improving overnight BP regulation [116]. Researchers investigated if the nighttime administration of one or more BP-lowering medications may regulate BP and minimize CV risk. After a follow-up of 5.6 years, individuals using one or more BP-lowering medications at bedtime had a lower overnight BP and a decreased incidence of nondipping/reversed dipping BP profile than patients taking all medications in the morning. Significantly, better nocturnal BP management was related to a significant reduction (60%) in the relative risk of CV events. It should be emphasized, however, that information on therapies intended to restore the BP circadian rhythm still needs to be improved and has been supplied by just a few research projects. The high BP readings in reversed dipping are considered dangerous compared to the increased daytime BP values [101]. Further research is therefore required on the primary traits, clinical implications, and prognostic implications of reverse dipping and preserving dipping patterns.

Reporters showed that reversed dipping BP profiles were more prevalent in the elderly, smokers, males, and a history of CVD [117]. Pressioni Arteriose Monitorate E Loro Associazioni (PAMELA) study revealed a similar prevalence [118]. Four European studies reported in a Belgian ABPM database that about 11% of the population had an inverted BP profile [119]. Recent research on untreated essential HTN patients (26% of whom had type 2 DM) revealed that, on average, nocturnal systolic BP values were higher than daytime values in almost one-fourth of patients (24%) [120]. It was learned that older age, type 2 diabetes, higher low-density lipoprotein cholesterol, and triglyceride concentrations were related to an inverted dipping BP pattern. It was observed that the greatest incidence of reversed dipping BP profile (36%) was in treated patients with essential HTN [98]. In Japanese research, reverse sinking BP prevalence was roughly 9% [121]. In the AASK trial, the reverse dipping HTN pattern was 39%, mostly due to the older age of the individuals included in the study (>60 years) [109].

The Chronic Kidney Disease Japan Cohort research revealed that 15.5% of CKD patients had elevated nighttime BP [122]. A greater prevalence of high BP has been observed in patients with stages 4 and 5 CKD compared to stage 3 and in patients with diabetes compared to non-diabetes. About 13% of those candidates were reverse dippers; they also had more moderate to severe obstructive sleep apnea incidence than other groups (13.5 vs. 9.0 vs. 7.5% in non-dippers and dippers, respectively) [123]. This development was associated with an increase in BP dippers [124]. Lastly, it is unknown if and to what degree the reverse dipper BP pattern constitutes a persistent clinical feature across time and whether it will cause persistent HTN. Hence, additional research is required to further study these issues.

White coat hypertension

Usually, the measurement of BP in a healthcare facility leads to higher readings for people who are generally not used to the place and/or medical professionals. Around 10-30% of patients with high BP values are inappropriately diagnosed with HTN. Although they do not have HTN, they have what is known as white coat HTN (WCHTN). The overall prevalence of WCHTN is about 10-15% in the general population and reported in about 30% in high BP readings at the clinic [125,126]. The reported prevalence of WCHTN differs according to the criteria for diagnosing this type of HTN. For instance, Zhu et al. reported a WCHTN in 5.1% of the Chinese population [127]. In a study, using the criteria of daytime, 24-h, or all ambulatory periods, the prevalence of WCHTN in the untreated group was found to be 41.3%, 35.2%, and 26.1%, respectively. In the treated group, the prevalence figures were 45.8% (daytime), 38.9% (24-h), and 27.2% (all ambulatory periods) - in all three categories of respective criteria [128]. This type of HTN is diagnosed or excluded using ABPM. WCHTN may cause people whose BP is genuinely normal to be misclassified when clinic-BP readings are used. Yet, there are still debates regarding WCHTN and its significance [129-131].

Thomas Pickering has introduced the term “white-coat hypertension”, describing people who did not take medicine for their high BP. These people have high office BP readings, but their BP readings are normal when monitored by the ABPM method. These people would have a low risk of CV events [132]. The recent European guidelines suggest a different definition of WCHTN, which includes people with systolic/diastolic BP readings of < 140/90 mmHg in the clinic and 24-hour ABPM of < 130/80 mmHg [133]. Current recommendations do not advise therapy; nevertheless, a new study indicates that this condition may still signify that overt HTN may develop [134]. Reported evidence claims harmful consequences from inappropriate antihypertensive medication if WCHTN patients have out-of-office BP readings at or below the target BP [135,136]. However, in patients aged > 80 years, WCHTN treatment is advisable to reduce CV and neurovascular events [137]. Instead, patients should keep up with their extensive lifestyle changes and efforts to lower their CV risk by controlling dyslipidemia and diabetes, which are usually present simultaneously. American College of Cardiology (ACC) and AHA guidelines describe WCHTN as an office BP of <130/80 mmHg but >160/100 mmHg, as well as a daytime BP of <130/80 mmHg on ABPM or home BP monitoring (HBPM) [6]. Conversely, European recommendations use office BP threshold levels of 140/90 mmHg [28]. In most known research, the cutoff values are 140/90 mmHg for office BP and 135/85 mmHg for out-of-office BP.

Guidelines advise using ABPM or HBPM for out-of-office BP monitoring to diagnose doubtful HTNs, including identifying white-coat HTNs. Those with suspected WCHTN should have the diagnosis verified within 3-6 months after their initial high BP reading, advisably by the ABPM method. Also, they should have ABPM or HBPM every year to see if persistent high BP is developing [133]. On the other hand, the Canadian diagnostic algorithm advises doing HBPM, or ABPM, after the first visit high BP record to recognize WCHTN patients as early as possible [138].

Automated self-office BP recording may reduce the late diagnosis of WCHTN and the number of people who require long hours of BP measurements [138]. One way to focus on out-of-office BP evaluation is to examine how BP readings change for one clinic visit. This opinion was reported as a great method to predict differences between BP results in the clinic and at home [139]. This would allow doctors to advise patients suspected of having WCHTN to use out-of-office methods more frequently. When used in combination with ABPM rather than in place of it, HBPM may be most helpful for identifying WCHTN because of its high specificity; however, it has poor sensitivity for WCHTN diagnosis [140]. A deep breathing test might be used as another strategy to target or minimize the usage of outside BP monitoring. In a clinical study, patients are unlikely to have WCHTN identified using ABPM if their systolic BP fell by 15% or less following 30 seconds of deep breathing [141].

Although the ABPM or HBPM recording may increase the service cost, it can effectively reduce the long-term treatment costs when the office BP reading is doubtful [142]. A model based on trial data predicted medical cost savings of US$ 1.56 million per 1000 patients over five years with HBPM included in the diagnosis of HTN [143]. According to a second examination of the same data, the widespread use of HBPM might lower Japan's medical expenses associated with HTN by US$ 9.3 million [144].

Pathogenesis of albuminuria in white coat hypertension

In recent years, the clinical pathogenicity of WCHTN itself has been demonstrated [145]; however, the mechanism by which WCHTN induces an increase in CV events remains unexplained [35,146]. In contrast, several potential processes may explain why WCHTN was related to albuminuria. WCHTN was characterized by high BP readings in the doctor's office, implying that WCHTN patients had supersensitive sympathetic nerve activity. As evaluated by microneurography [147], individuals with WCHTN showed considerably more SNS activity than normotensive individuals [148]. Patients with WCHTN showed a tachycardia to bradycardia variability ratio than those with normotensive. The WCHTN group's BP response to mental stress was significantly larger than the normotensive [149]. In the first mechanism, it was considered that this increased sympathetic activity was the main risk factor for CVD, CKD, and mortality [150,151], suggesting that this sympathetic system hypersensitivity may cause WCHTN. According to research by Ohasama, patients with WCHTN often suffer from metabolic syndromes [152]. It is also thought that metabolic syndromes due to the caused endothelial dysfunction may lead to albuminuria, increasing CVD and death. A third possible mechanism of albuminuria in WCHTN is that these individuals are more likely to acquire secondary HTN than those with normal BP [153]. Sustained HTN is a chief risk factor for CVD, CKD, and albumin in the urine. Lastly, it is plausible that WCHTN directly causes albuminuria, but no corroborating data. Moreover, whether albuminuria in WCHTN indicates development and CKD advancement remains to be determined. Further research is required to assess the significance of albuminuria in WCHTN patients as a predictor for renal damage.

Masked hypertension

Since Thomas Pickering in 2002 announced the term ‘masked HTN’, it gained widespread attention [154]. The frequency of masked HTN ranges between 8-20% in untreated adults and almost 61% in treated adults [155,156]. When the BP readings are normal in a health service or clinic facility, but BP readings are high by ABPM or HBPM, then masked HTN should be considered as a diagnosis. There is a need to encourage the usage of ABPM or home BP measuring devices when BP readings are in the normal range inside the clinic, but BP readings are high outside. However, incidental BP measurements may occasionally reveal this condition, and an explanation is needed when masked HTN is discovered during sleep. Obstructive sleep apnea can cause high systolic BPs, and essential HTN can cause gradually increasing pressures, altering the BP dipping pattern. The nighttime drop in BP can disappear due to dreaming, which may cause nighttime narrow systolic HTN peaks.

Approximately 20% of those missed individuals who go untreated may develop masked HTN. Masked HTN increases carotid atherosclerosis risk and the LV muscle mass index compared with normotensive persons [157]. A Japanese study reported a decline in EGFR in masked HTN patients [158]. Due to these reported CV and renal complications, it was recommended to treat masked HTN [159]. Antihypertensive drugs should be used to treat this type of HTN since they may increase HTN complications risks.

Sustained (persistent) hypertension

A subject with high BP readings both during home monitoring and at health facilities is labeled as having sustained HTN, which is highly linked to an increased risk of renal and cardiac damage, necessitating early anti-HTN therapy initiation.

Kidney and blood pressure variation

Although some researchers have indicated that nocturnal BP predicts CVD and death [160], others did not support this [161]. The loss of a normal drop in nocturnal BP in hypertensive patients is linked with target organ dysfunction such as the left ventricle and kidneys [69], microalbuminuria [162], cerebrovascular [163], and CV events [164]. Few studies in normotensive patients focused on urine albumin excretion, examining the association between the diurnal rhythm of BP and renal outcomes. Previous research in normotensive people revealed a weak correlation between the LV mass index and dipping BP profile [165] but not with urine albumin excretion [166]. Borrelli et al. concluded that high systolic BP recorded by ABPM or loss of nocturnal BP dipping is linked to higher rates of CVD and CKD progression, especially in CKD patients [33]. The nondipping BP profile is more common in CKD patients due to increased SNS activity [167]; the non-dipper profile raises the risk of renal disease [77]. The loss of kidney function and the prediction factors and measures still need to be clarified through further studies.

The currently available data revealed no discernible difference in urine protein content between nondippers and dippers with low BP. Previous research on progressive reduction in the estimated glomerular filtration rate (eGFR) showed that nighttime BP was the best indicator of renal disease development [113]. In a recent study reported in 1,042 hypertensive patients after almost six years, the eGFR decreased by -0.23 to -0.20 ml/min/year, as the decline in the rates of nighttime systolic, diastolic BP, and mean arterial pressure raised by 1% (P < 0.001). Although the average nighttime BP in the nondipper low BP group was more significant than in the dipper low BP group, the eGFR drop rate was not different among these groups. The recent findings did not confirm an exact figure for this; however, they thought that the nocturnal BP threshold is not a significant factor for CKD development. They concluded that nighttime BP could be utilized as a kidney injury marker in HTN patients [168].

Due to a need for more robust reported data, it is still being determined whether reversing non-dipper to dipper BP patterns by BP-lowering agents to potentiate renoprotective effects is significantly effective. Many clinical studies have shown that the normalization of BP’s diurnal rhythm by anti-hypertensives improves proteinuria in early diabetic nephropathy [113] and lowers CV events risk [169]. Crespo et al. demonstrated that bedtime HTN therapy for CKD patients decreased the mean nighttime BP and the proportion of non-dippers [170]. Kado et al. reported that even though it is an observational result, switching normotensive patients’ BP profile from a nondipper to a dipper may not be advantageous for preventing the course of kidney disease progression [171]. Another study reported that ABPM provides extra information for kidney and CV outcomes in hypertensive CKD prediction than multiple clinic BP measures [32]. Because ABPM itself interrupts the sleeping state and lowers daytime activity, which results in greater nocturnal BP and lowers daytime BP than typical, the producibility of ABPM may sometimes be limited. Even at intervals of many weeks, previous research revealed that some people had a distinct circadian BP profile in serial ABPM [71]. The 48-hour ABPM may be more accurate in representing the BP profile [172], although it is less practical for routine clinical use. The long-term effect of BP control on the drop in the eGFR is also unknown since no studies for evaluation of BP control used ABPM for BP readings during the 48-hour ABPM-observational period was observed. A clinical review concluded a significant correlation between the baseline eGFR and annual eGFR drop during BP-reduction therapy. Additionally, in the same review, they reported that the 20% reduction of the eGFR following BP reduction might be accepted and probably extended to 46% depending upon the reduction achieved [173].

The likelihood of developing kidney disease is more in nondipper than the dipper-hypertensive-CKD patients. Kado et al. reported that normotensive nondipping CKD patients have a lesser risk of rapid eGFR drop [166]. Additionally, a previous cohort analysis showed that an elevated risk of ESRD was linked to an eGFR drop for a one-year follow-up [174]. These findings imply that maintaining eGFR depends more on controlling BP than maintaining its circadian rhythm. Clarifying the impact of the diurnal regularity of BP on renal disease prognosis in normotensives, certain research parameters would be needed for high-powered research such as a higher number of research participants, inclusive population, and a long observation time.

Borrelli et al. concluded that high systolic BP measured by ABPM or nocturnal BP dipping loss is linked to higher CVD and CKD progression rates, especially in CKD patients [33]. A non-dipping BP profile is more common in CKD patients [175]; the non-dipper profile raises the risk of renal disease [167]. Nevertheless, it takes a great deal of effort to normalize both the actual BP value and the BP rhythm throughout the day.

Few studies focused on the urine albumin excretion while examining the association between renal outcomes and BP rhythm in normotensive patients. Previous research in normotensive population revealed a weak correlation between dipping BP profiles and left ventricular mass index [165] but not with urine albumin excretion [166]. A recent study conducted on adolescents found that there was no conclusive evidence to suggest a link between night-time BP dipping and cardiac structure changes. However, it was reported that measuring BP variability could be helpful in assessing the risk of CV remodeling, even in adolescents with BP within the physiological range [176]. The loss of kidney function still needs to be clarified and studied further.

The current data revealed no discernible difference in protein urine content between non-dippers and dippers with low BP. Previous research on progressive eGFR reduction showed that nighttime BP was the best indicator of development of renal disease [113]. In a recent study reported in 1,042 hypertensive patients after almost six years, the eGFR decreased by -0.23 to -0.20 ml/min/year, as the nighttime systolic, diastolic BP, and mean BP decline rates raised by 1% (P < 0.001). They concluded that the nighttime BP could be utilized as a marker of kidney injury in HTN patients [168]. Although the average nighttime BP in the nondipper low BP group was more significant than in the dipper low BP group in the current investigation, there was no discernible difference in the rate of eGFR fall between these groups. The recent findings did not set an exact figure for this, but they suggest that nocturnal BP and below the BP threshold are not important factors in developing kidney disease [168].

The normalization of the diurnal rhythm of the BP by administering anti-hypertensives has been shown in many clinical studies to improve proteinuria in early diabetic nephropathy [113] and lower the risk of CVDs [174]. Crespo et al. demonstrated that bedtime HTN therapy in CKD patients decreased the mean of night BP and the proportion of nondippers [170]. Kado et al. reported that even though it is an observational result, switching a normotensive nondipper to a dipper may not be advantageous for preventing the course of renal disease progression [171]. Another study reported that ABPM provides extra information for kidney and CV outcomes in hypertensive CKD prediction than multiple clinic BP measures [32].

Because ABPM interrupts the sleeping state and lowers daytime activity, which results in greater nocturnal BP and lowers daytime BP than typical, the repeatability of ABPM may sometimes be limited. Even at intervals of many weeks, previous research revealed that some people had a distinct circadian BP profile in serial ABPM [71]. The 48-hour-ABPM may more accurately represent the BP profile [172], although it is less practical for routine clinical use.

The low incidence of individuals who exhibit dipping BP patterns is another drawback, possibly due to the small sample size that impacted the significance of BP variations in the reported data. Furthermore, even though the nondipping profile was not a factor for the development of kidney disease in normotensive individuals, there is a precise value of the BP risk threshold. Prospective interventional research with several ABPM tests is required to set a target BP. Uncertainty persists on the best time to take BP readings that accurately represent the declining BP trend and the average readings day and night. Previous research showed that self-home BP recording is an alternate tool for forecasting end-organ damage and reasonably correlating with ABPM, and both are superior to office BP monitoring [9].

Kado et al. reported that a nondipping BP profile in normotensive CKD patients does not indicate a risk for a quick fall in eGFR [166]. The nondipping BP profile increases the likelihood of developing kidney disease in hypertensive CKD patients more than in dipping BP. Additionally, a prior community-based cohort analysis showed that an elevated risk of ESRD was linked to an eGFR drop for a one-year follow-up [174]. These findings infer that maintaining eGFR depends more on controlling BP than maintaining its circadian rhythm. More participants and a long observation time are necessary to assess the impact of the diurnal regularity of BP on the renal disease prognosis in normotensives.

## Conclusions

Ambulatory blood pressure monitoring has impacted blood pressure classification and encouraged research on hypertension patterns, effects, and types. Physical activity, subjective sleep quality, and consecutive day recordings improve the reproducibility of blood pressure measurement. Volume-related variables may play a role in abnormal blood pressure patterns. Nondipping hypertension is linked to salt sensitivity, renal function impairment, sodium retention, and mineralocorticoid-induced hypertension; however, further studies are required to certain the relationship.

Reversing the BP dipping pattern may have poor CV prognostic effects, although there is conflicting evidence; hence, more research is needed on treatment approaches that restore circadian BP rhythms and the long-term effect of circadian rhythms' restoration.
